# Impact of coronary collateralization on long-term clinical outcomes in type 2 diabetic patients after successful recanalization of chronic total occlusion

**DOI:** 10.1186/s12933-020-01033-4

**Published:** 2020-05-11

**Authors:** Zhen Kun Yang, Ying Shen, Yang Dai, Xiao Qun Wang, Jian Hu, Feng Hua Ding, Rui Yan Zhang, Lin Lu, Wei Feng Shen

**Affiliations:** 1 Department of Cardiology, Rui Jin Hospital, Shanghai Jiaotong University School of Medicine, Shanghai, 200025 People’s Republic of China; 2 Institute of Cardiovascular Diseases, Shanghai Jiaotong University School of Medicine, Shanghai, 200025 People’s Republic of China

**Keywords:** Chronic total occlusion, Diabetes mellitus, Coronary collateral circulation, Percutaneous coronary intervention, Prognosis

## Abstract

**Background:**

To assess the prognostic role of coronary collaterals in patients with type 2 diabetes mellitus (T2DM) after successful percutaneous coronary intervention (PCI) for chronic total occlusion (CTO).

**Methods:**

Coronary collateralization was graded according to Rentrop scoring system in 198 type 2 diabetic patients and 335 non-diabetics with stable angina undergoing PCI for at least one CTO lesion. Left ventricular ejection fraction (LVEF) was determined and major adverse cardio-cerebral events (MACCE) were recorded during follow-up.

**Results:**

Poor collateralization was more common in patients with T2DM than in non-diabetics (40% vs 29%, p = 0.008). At 13.5 ± 4.1 months, the rate of composite MACCE (17.3% vs 27.6%, p = 0.034) and repeat revascularization (15.2% vs 25.5%, p = 0.026) was lower and the increase in LVEF (3.10% vs 1.80%, p = 0.024) was greater in patients with good collaterals than in those with poor collaterals for non-diabetic group. The associations were in the same direction for T2DM group (35% vs 44%; 30% vs 36%; 2.14% vs 1.65%, respectively) with a higher all-cause mortality in diabetic patients with poor collaterals (p = 0.034). Multivariable Cox proportional hazards analysis showed that coronary collateralization was an independent factor for time to MACCE (HR 2.155,95% CI 1.290–3.599, p = 0.003) and repeat revascularization (HR 2.326, 95% CI 1.357–3.986, p = 0.002) in non-diabetic patients, but did not enter the model in those with T2DM.

**Conclusions:**

T2DM is associated with reduced coronary collateralization. The effects of the status of coronary collateralization on long-term clinical outcomes and left ventricular function appear to be similar in size in type 2 diabetic patients and non-diabetics after successful recanalization of CTO.

## Background

Chronic total occlusion (CTO) occurs in 5–10% of patients with significant coronary artery disease undergoing routine coronary angiography, which is more common in those with diabetes mellitus [1, 2]. Both randomized trials and observational studies have demonstrated that successful revascularization of chronic totally occluded lesions accomplished by percutaneous coronary intervention (PCI) or coronary artery bypass grafting is associated with a number of clinical benefits, such as anginal symptom relief, improved quality of life and ventricular function, and decreased mortality when compared to CTO patients whose recanalization was failed or those who received optimal medical treatment only [3–7]. Therefore, revascularization is recommended as an initial therapeutic modality in patients with CTO by current guidelines [8]. Recently, with the improvement in dedicated devices, technical strategies and interventional skills, the overall success rate of PCI for CTO (CTO-PCI) has been dramatically increased [9].

Diabetes mellitus is regarded as a coronary heart disease risk equivalent and an important factor when planning treatment strategies for coronary artery disease as well as evaluating clinical outcomes after PCI [10, 11]. Patients with type 2 diabetes mellitus (T2DM) often have an elevated incidence of CTO (approximately 30–40% in the registry) [12, 13]. In the setting of complete coronary obstruction, blood supply to the distal myocardium is solely from collateral vessels. The formation and maturation of coronary collateral circulation is an adaptive physiologic response, and well-formed collaterals may minimize infarcted area and improve ventricular function and survival compared with patients lacking a well-developed collateral network [14, 15]. However, the prognostic role of collaterals in patients with coronary artery disease remains controversial. In myocardial infarction patients with acute coronary occlusion, previous studies yielded mixed results with some showing improved clinical outcomes in patients with angiographic evidence of collaterals [16–19] and others revealing either no difference [20–22] or worse outcomes [23]. In stable coronary artery disease patients with CTO, while T2DM is strongly associated with reduced coronary collateral formation [24–26], few published studies have focused on the impact of coronary collaterals on clinical prognosis, especially for those with T2DM after CTO-PCI [6].

Therefore, we conducted a prospective, observational study to examine the effect of coronary collateralization on long-term clinical outcomes in type 2 diabetic and non-diabetic patients with stable coronary artery disease after successful CTO-PCI.

## Methods

The protocol was approved by the Shanghai Jiao Tong University ethic committee and conducted in accordance with the Declaration of Helsinki. All patients gave written informed consent.

### Study population

A total of 779 consecutive patients with stable angina who were attempted to undergo CTO-PCI of at least one major epicardial coronary artery between January 2016 and December 2018 were recruited from the database of Shanghai Rui Jin Hospital PCI Outcome Program. This program utilizes clinical and angiographic information for various cardiovascular diseases to estimate risk-adjusted outcomes. Data on demographics, clinical and angiographic features, left ventricular function determined by two-dimensional echocardiography according to modified Simpson’s rule, and in-hospital management were collected retrospectively, whereas clinical outcomes during follow-up were identified prospectively.

**Fig. 1:**
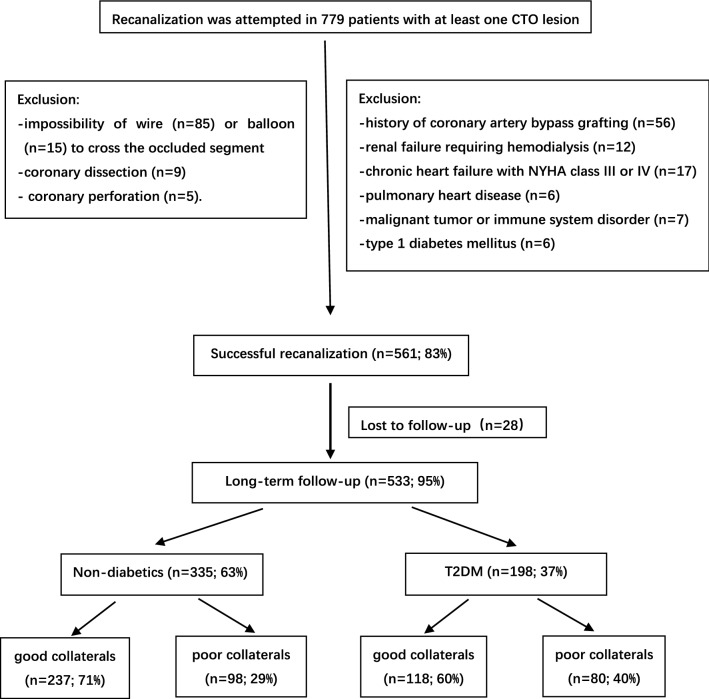
Flowchart patient enrollment. *CTO* chronic total occlusion, *T2DM* type 2 diabetes mellitus

For the purpose of this study, 98 patients were excluded because of a history of coronary artery bypass grafting (n = 56), renal failure requiring hemodialysis (n = 12), chronic heart failure with NYHA class III or IV (n = 17), pulmonary heart disease (n = 6) and malignant tumor or immune system disorders (n = 7), as these conditions could influence collateral formation. Patients with type 1 diabetes (n = 6) were excluded by measurement of C-peptide level. In the 675 eligible patients, CTO-PCI was successful in 561 patients (83%). The main causes for failed procedure included impossibility of wire (n = 85) or balloon (n = 15) to cross the occluded segment and major complications (coronary dissection: n = 9; coronary perforation: n = 5). We also excluded additional 28 (5%) patients who were lost to follow up. Thus, the remaining 533 patients were enrolled in the final analyses. Among them, 198 patients (37%) had T2DM and 335 (63%) were non-diabetics (Fig. 1).

CTO was defined as those occluded arteries with a documented duration of occlusion of at least 3 months with absolutely no flow through the lesion (TIMI grade 0) [27]. Estimation of the duration of coronary occlusion was based on the first onset of an abrupt worsening of existing angina, a history of myocardial infarction in the target vessel territory, or information obtained from a previous angiogram. Stable angina was diagnosed according to the criteria recommended by the American College of Cardiology/American Heart Association [28]. T2DM was defined as a fasting glucose level > 126 mg/dL or glycated hemoglobin A1c concentration greater than 6.5% assessed at least once, or the current use of oral hypoglycemic agents or insulin [29].

### Coronary intervention procedure

Coronary angiography and intervention were performed with standard techniques using 6F or 7F guiding catheters via the radial or femoral approach and drug-eluting stent implantation as the default strategy (> 95% cases). Before the procedure, all patients received loading dose of aspirin (300 mg/d) and clopidogrel (300 mg) or ticagrelor (180 mg). During the procedure, an intravenous bolus of heparin (70–100 IU/kg) was given, but the use of glycoprotein IIb/IIIa inhibitors was at the operator’s discretion. CTO-PCI was performed using contemporary techniques such as bilateral injection; specialized hydrophilic, tapered tip, and stiff wires; parallel wires; microcatheters; and retrograde approach. The choice of guidewires, balloons, and drug-eluting stent type was left to the discretion of the operators. After the procedure, clopidogrel (75 mg/day) or ticagrelor (90 mg, twice daily) was prescribed for at least 12 months, and aspirin (100 mg/day) was continued indefinitely. After discharge, all patients were encouraged to take guideline- recommended medications including statins, angiotensin-converting enzyme inhibitors and β-blockers unless contraindicated, and to receive repeat coronary angiography at 12 months during follow-up.

Technical success was defined as a residual stenosis of < 20% and restoration of TIMI grade 3 flow. Procedural success was defined as technical success without death, myocardial infarction, or emergency coronary bypass grafting. Complete revascularization was defined as restoration of TIMI grade 3 flow with residual stenosis < 20% in all three major coronary arteries and their branches (diameter ≥ 2.0 mm).

### Coronary collateral scoring

The degree of coronary collaterals supplying the distal aspect of a total occlusion from the contra-lateral vessel was graded according to Rentrop classification: 0 = no visible filling of any collateral channel; 1 = filling of side branches of the artery to be perfused by collateral vessels without visualization of epicardial segment; 2 = partially filling of the epicardial artery by collateral vessels; 3 = complete filling of the epicardial artery by collateral vessels [30]. Patients were categorized into poor (grade 0 or 1) or good (grade 2 or 3) coronary collateralization group. All angiograms were viewed by the two observers blinded to the other observers’ findings, and the agreement of the assessment of coronary artery disease severity and collateral classification between the two observers was 98% and 97%, respectively [31]. Any difference in interpretation was resolved by a third reviewer. For those with more than one total coronary occlusion, the vessel with the highest collateral grade was chosen for analysis.

### Study endpoints

The primary study endpoint was the occurrence of composite major adverse cardio-cerebral events (MACCE) during follow-up, including all-cause death, cardiac death, non-fatal myocardial infarction, repeat revascularization, and non-fatal stroke. All-cause death was defined as any post-procedure death, and the cause was considered cardiac unless a definite non-cardiac cause was established. Myocardial infarction was defined as recurrent symptoms with new electrocardiographic changes compatible with myocardial infarction or cardiac marker level at least twice the upper limit of normal. Repeat revascularization defined as any revascularization of either the target or non-target vessels with PCI or coronary artery bypass grafting was performed on patients with severe in-stent restenosis or new coronary lesions (luminal diameter narrowing > 70%). In-stent restenosis was defined as recurrence of lumen diameter reduction > 50% within the stent or 5 mm proximal or distal segment adjacent to the stent at follow-up angiography [32]. Atherosclerotic lesion progression was diagnosed if one of the following criteria was met: (1) ≥ 20% diameter reduction of a pre-existing stenosis > 50%; (2) ≥ 30% diameter reduction of a stenosis < 50%; (3) progression of any stenosis to total occlusion, or (4) development of a new stenosis > 50% in a previously normal segment [33]. The secondary study endpoint was the change in left ventricular ejection fraction (LVEF) determined by two-dimensional echocardiography using modified Simpson’s method.

### Statistical analysis

Continuous variables are expressed as mean ± standard deviation (SD) and categorical data as percentages. Two-side Student’s t test was used to compare continuous variables, and Pearson Chi square statistics was used to compare categorical values. The rate of composite MACCE and repeat revascularization were compared by calculating risk ratio with 95% confidence intervals (CIs). Cumulative rate of individual and composite outcomes was estimated using the Kaplan–Meier methods and compared with the log-rank test. Multivariable models were built by stepwise variable selection, and covariates with p < 0.10 level on univariable analysis or clinically relevant were considered candidate variables. Adjusted hazard ratios were compared by Cox regression based on: age, gender, risk factors for coronary artery disease (current smoking, hypertension, hypercholesterolemia, and diabetes), extent of coronary artery disease (categorized as 1-, 2-, or 3-vessel disease), collateral classification, glomerular filtration rate, pre-procedural LVEF, and completeness of revascularization. A probability level of p < 0.05 was considered significant. All analyses were performed using the software package SPSS, version13 (SPSS Inc, Chicago, IL, USA).

## Results

### Clinical and angiographic features

Compared with non-diabetic patients, those with T2DM were more males in gender distribution, and had higher percentage of hypertension, multivessel disease and poorer coronary collaterals, and lower LVEF (Table 1). Univariable regression analysis showed that presence of T2DM was the only determinant of poor coronary collateralization (r = 0.114, p = 0.008).

**Table 1:** Clinical, angiographic and procedural characteristics of patients stratified according to T2DM and coronary collateral flow

Variable	All patients (n = 533)	Non-diabetes (n = 335) (63%)				Diabetes (n = 198) (37%)				p value (non-diabetes vs diabetes)
Rentrop coronary collateral classification		All	Good CC (n = 237) (71%)	Poor CC (n = 98) (29%)	p value	All	Good CC (n = 118) (60%)	Poor CC (n = 80) (40%)	p value	0.008
Age (years)	62.61 ± 10.99	62.37 ± 11.35	61.66 ± 11.10	64.08 ± 11.81	0.075	63.02 ± 10.36	63.26 ± 10.13	62.65 ± 10.75	0.684	0.511
Men	427 (80%)	281 (84%)	202 (85%)	79 (81%)	0.296	146 (74%)	87 (74%)	59 (74%)	0.997	0.005
CAD risk factors										
Hypertension	374 (70%)	222 (66%)	149 (63%)	73 (74%)	0.041	152 (77%)	96 (81%)	56 (70%)	0.063	0.01
Diabetes										
Current smoking	195 (37%)	119 (36%)	87 (37%)	32 (33%)	0.48	76 (38%)	43 (36%)	33 (41%)	0.495	0.508
Hypercholesterolemia	69 (13%)	37 (11%)	25 (11%)	12 (12%)	0.652	32 (16%)	18 (15%)	14 (18%)	0.674	0.089
Body mass index (kg/m2)	25.56 ± 3.27	25.37 ± 3.12	25.35 ± 3.03	25.42 ± 3.34	0.861	25.87 ± 3.49	25.79 ± 3.26	25.98 ± 3.84	0.73	0.09
Glomerular filtration rate (ml/min)	84.61 ± 19.28	85.12 ± 17.42	85.97 ± 17.24	83.05 ± 17.78	0.163	83.76 ± 22.08	84.29 ± 21.22	82.98 ± 23.41	0.684	0.432
LVEF (%),at baseline	52.39 ± 7.38	53.00 ± 7.27	53.52 ± 6.81	51.74 ± 8.18	0.042	51.36 ± 7.46	51.70 ± 7.48	50.85 ± 7.44	0.431	0.013
Severity of coronary artery disease					0.967				0.751	0.006
1	111 (21%)	82 (24%)	58 (24%)	24 (24%)		29 (15%)	19 (16%)	10 (12%)		
2	214 (40%)	137 (41%)	96 (41%)	41 (42%)		77 (39%)	46 (39%)	31 (39%)		
3	208 (39%)	116 (35%)	83 (35%)	33 (34%)		92 (46%)	53 (45%)	39 (49%)		
Number of CTO lesions recanalized	560	350	250	100		210	123	87		
Location of CTO lesion					0.001				< 0.001	0.864
Left anterior descending	189 (34%)	121 (35%)	72 (29%)	49 (49%)		68 (32%)	31 (25%)	37 (42%)		
Left circumflex artery	82 (15%)	51 (14%)	37 (14%)	14 (14%)		31 (15%)	13 (11%)	18 (21%)		
Right coronary artery	289 (51%)	178 (51%)	141 (57%)	37 (37%)		111 (53%)	79 (64%)	32 (37%)		
Number of stents for CTO vessel	2.01 ± 0.93	2.03 ± 0.93	2.08 ± 0.96	1.90 ± 0.85	0.109	1.99 ± 0.92	2.08 ± 0.90	1.85 ± 0.93	0.073	0.621
Complete revascularization (%)	433 (81%)	268 (80%)	187 (79%)	81 (83%)	0.435	165 (83%)	98 (83%)	67 (84%)	0.897	0.341
Medications										
Oral antiplatelet agent	533 (100%)	335 (100%)	237 (100%)	98 (100%)	–	198 (100%)	118 (100%)	80 (100%)	–	–
Statin	486 (91%)	301 (90%)	217 (92%)	84 (86%)	0.107	185 (93%)	107 (91%)	78 (97%)	0.057	0.159
β-blocker	439 (82%)	276 (82%)	192 (81%)	84 (86%)	0.304	163 (82%)	93 (79%)	70 (87%)	0.116	0.985
ACEI or ARB	320 (60%)	206 (61%)	154 (65%)	52 (53%)	0.041	114 (58%)	66 (56%)	48 (60%)	0.570	0.372
Insulin	25 (5%)	0	0	0	–	25 (13%)	15 (13%)	10 (12%)	0.965	–
Oral antidiabetic drug	173 (32%)	0	0	0	–	173 (87%)	103 (87%)	70 (88%)	0.965	–

*T2DM* type 2 diabetes mellitus, *CC* coronary collateralization, *CAD* coronary artery disease, *LVEF* left ventricular ejection fraction, *CTO* chronic total occlusion, *ACEI* angiotensin-converting enzyme inhibitor, *ARB* angiotensin receptor blocker

During PCI procedure, a total of 560 CTO lesions were successfully recanalized with drug-eluting stent implantation. There were no differences in location of CTO, stent number for CTO lesion and the rate of complete revascularization between patients with and without T2DM except that those with T2DM had higher proportion of multivessel disease (p = 0.006) (Table 1).

### Long-term clinical outcomes

During a mean of 13.5 ± 4.1 months of follow-up, 8 patients died (1.5%); 5 of them had cardiac death (good collaterals in 1 patient [0.3%] and poor collaterals in 4 patients [2.2%], p = 0.026). and other 3 died of respiratory failure due to pulmonary infection (n = 1) or cerebrovascular accidents (n = 2). All patients received coronary angiography during follow-up, and 125 (23.5%) of them underwent repeat revascularization because of severe in-stent restenosis or new lesions and disease progression. Overall, the rate of composite MACCE (38% vs 19.4%, p < 0.001) and repeat revascularization (32% vs 18.2%, p < 0.001) was higher in type 2 diabetic patients compared to non-diabetics.

**Fig. 2:**
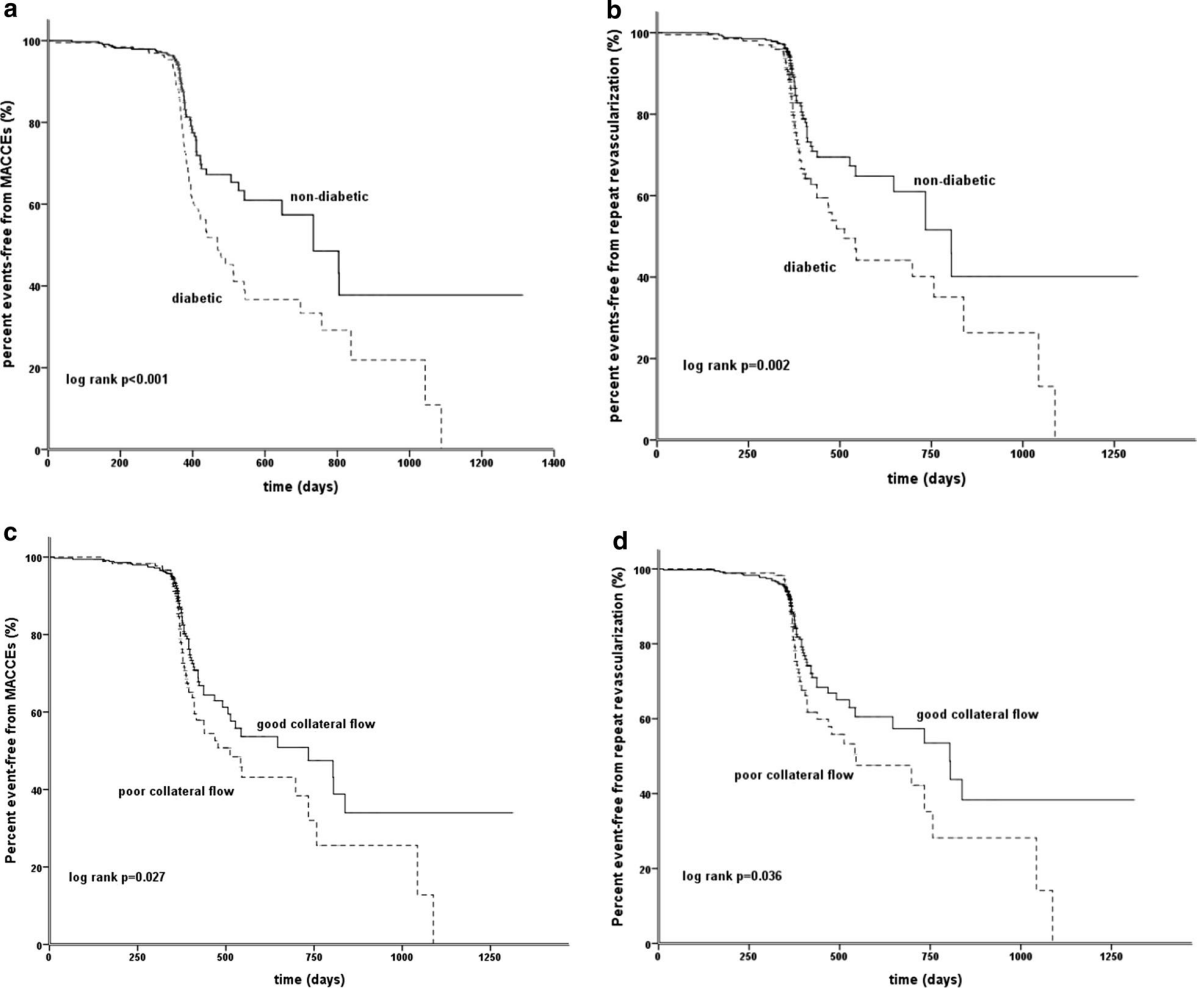
Kaplan-Meier curves. Percent event-free from MACCE and repeat revascularization in non-diabetic and type 2 diabetic patients** a**,** b** and in those with good and poor coronary collateral circulation** c**,** d**. MACCE major adverse cardio-cerebral events

For non-diabetic patients, poor collateralization was associated with more in-stent restenosis (14.3% vs 6.3%, p = 0.018) and repeat revascularization (25.5% vs 15.2%, p = 0.026), leading to a higher rate of composite MACCE (27.6% vs 17.3%, p = 0.034). In contrast, for type 2 diabetic patients, the rate of composite MACCE, in-stent restenosis and repeat revascularization did not differ irrespective of collateral status, except for a higher all-cause mortality in those with poor collateralization (p = 0.034) (Table 2). Kaplan–Meier curve analysis revealed a clear difference in percent event-free from MACCE or repeat revascularization according to the presence or absence of T2DM or Rentrop class of coronary collateralization (Fig. 2).

**Table 2:** Clinical outcomes stratified according to T2DM and coronary collateralization

Variable	All patients (n = 533)	Non-diabetes (n = 335) (63%)				Diabetes (n = 198) (37%)				p value (non-diabetes vs diabetes)
		All	Good CC (n = 237) (71%)	Poor CC (n = 98) (29%)	p value	All	Good CC (n = 118) (60%)	Poor CC (n = 80) (40%)	p value	
MACCEs (composite)	144 (27%)	68 (19.4%)	41 (17.3%)	27 (27.6%)	0.034	76 (38%)	41 (35%)	35 (44%)	0.201	< 0.001
All cause death	8 (1.5%)	5 (1.5%)	3 (1.3%)	2 (2.0%)	0.595	3 (1.5%)	0	3 (3.8%)	0.034	0.983
Cardiac death	5 (0.9%)	3 (0.9%)	1 (0.4%)	2 (2.0%)	0.152	2 (1.0%)	0	2 (2.5%)	0.084	0.895
Non-fatal MI	0	0	0	0	NS	0	0	0	NS	NS
Non-fatal stroke	17 (3.2%)	8 (2.4%)	7 (2.9%)	1 (1.0%)	0.292	9 (4.5%)	6 (5.1%)	3 (3.8%)	0.658	0.171
Repeat revascularization	125 (23.5%)	61 (18.2%)	36 (15.2%)	25 (25.5%)	0.026	64 (32%)	35 (30%)	29 (36%)	0.331	< 0.001
ISR of CTO lesion	65 (12.2%)	29 (8.7%)	15 (6.3%)	14 (14.3%)	0.018	36(18%)	20 (17%)	16 (20%)	0.585	0.001
New lesion or lesion progression	67 (12.6%)	33 (9.9%)	22 (9.3%)	11 (11.2%)	0.587	34 (17%)	16 (14%)	18 (23%)	0.102	0.014

*T2DM* type 2 diabetes mellitus, *CC* coronary collateralization, *MACCEs* major adverse cardio-cerebral events, *MI* myocardial infarction, *ISR* in-stent restenosis, *CTO* chronic total occlusion

### Left ventricular function

Baseline LVEF was higher in patients with good collaterals than that in those with poor collateral for non-diabetics but was similar between type 2 diabetic patients with good and poor collaterals. Successful CTO-PCI was associated with a mild increase in LVEF irrespective of the status of diabetes and coronary collateralization (all p < 0.001). The degree of increase in LVEF was significantly greater in patients with good collaterals than in those with poor collaterals for non-diabetic group (3.10% vs 1.80%, p = 0.024) but did not differ between patients with good and poor collaterals for T2DM group (Table 3).

**Table 3:** Left ventricular ejection fraction before CTO-PCI and during follow-up

Variable	All patients (n = 533)	Non-diabetes (n = 335) (63%)				Diabetes (n = 198) (37%)				p value (non-T2DM vs T2DM)
		All	Good CC (n = 237)	Poor CC (n = 98)	p value	All	Good CC (n = 118)	Poor CC (n = 80)	p value	
LVEF (%) at baseline	52.39 ± 7.38	53.00 ± 7.27	53.52 ± 6.81	51.74 ± 8.18	0.042	51.36 ± 7.46	51.70 ± 7.48	50.85 ± 7.44	0.431	0.013
LVEF (%) at follow-up	54.89 ± 6.54	55.72 ± 6.18	56.62 ± 5.73	53.54 ± 6.71	< 0.001	53.49 ± 6.89	54.17 ± 6.56	52.50 ± 7.29	0.095	< 0.001
ΔLVEF (%)	2.50 ± 4.63	2.72 ± 4.82	3.10 ± 4.75	1.80 ± 4.87	0.024	2.14 ± 4.29	2.47 ± 4.22	1.65 ± 4.37	0.19	0.163
p value (baseline vs follow-up)	< 0.001	< 0.001	< 0.001	< 0.001		< 0.001	< 0.001	< 0.001		

*T2DM* type 2 diabetes mellitus, *CC* coronary collateralization, *CTO* chronic total occlusion, *PCI* percutaneous coronary intervention, *LVEF* left ventricular ejection fraction, *ΔLVEF* change in eft ventricular ejection fraction

### Multivariate analysis

**Fig. 3:**
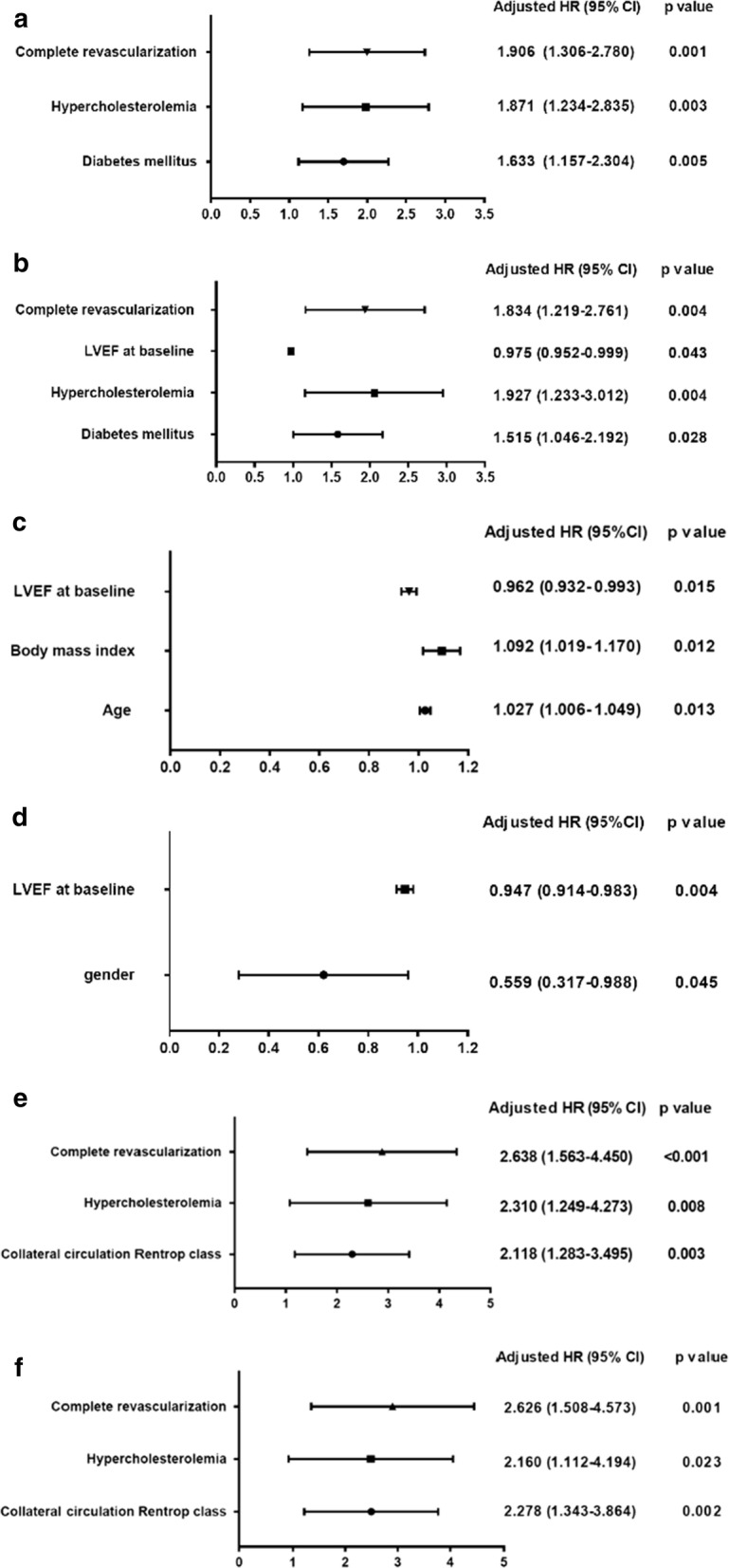
Independent predictors of MACCE and repeat revascularization in all patients **a**, **b**, diabetic **c**, **d** and non-diabetic patients **e**, **f** on multivariable analysis. *MACCE* major adverse cardio-cerebral events, *LVEF* left ventricular ejection fraction

Multivariable Cox proportional hazards regression analysis revealed that after adjusting for covariates with p < 0.10 level on univariable analysis (Table 4), including age, gender, risk factors for coronary artery disease (current smoking, hypertension, hypercholesterolemia, and diabetes), extent of coronary artery disease (categorized as 1-, 2-, or 3-vessel disease), collateral classification, glomerular filtration rate, pre-procedural LVEF, and completeness of revascularization, T2DM but not the status of coronary collateralization was independent factors for time to MACCE (HR 1.633,95% CI 1.157–2.304, p = 0.005) and repeat revascularization (HR 1.515, 95% CI 1.046–2.192, p = 0.028) (Additional file 1: Table S1). Further analysis showed that the status of coronary collateralization was an independent factor for time to MACCE (HR 2.118, 95% CI 1.283–3.495, p = 0.003) and repeat revascularization (HR 2.278, 95% CI 1.343–3.864, p = 0.002) in non-diabetic patients, but it did not enter the model in those with T2DM (Fig. 3; Additional file 2: Table S2; Additional file 3: Table S3).

**Table 4:** Predictors of MACCE and repeat revascularization in all patients, non-diabetic and diabetic patients on univariable analysis

Variable	All patients				Non-diabetes				Diabetes			
	MACCE		Repeat revascularization		MACCE		Repeat revascularization		MACCE		Repeat revascularization	
	Hazard ratio (95% CI)	p value	Hazard ratio (95% CI)	p value	Hazard ratio (95% CI)	p value	Hazard ratio (95% CI)	p value	Hazard ratio (95% CI)	p value	Hazard ratio (95% CI)	p value
Age	1.009 (0.994–1.025)	0.233	0.997 (0.981–1.013)	0.684	0.998 (0.977–1.020)	0.888	0.989 (0.967–1.012)	0.355	1.019 (0.998–1.041)	0.072	1.004 (0.981–1.028)	0.740
Gender	0.876 (0.593–1.294)	0.507	0.858 (0.565–1302)	0.471	1.421 (0.704–2.868)	0.327	1.670 (0.759–3.673)	0.202	0.733 (0.450–1.193)	0.211	0.640 (0.380–1.077)	0.093
Hypertension	1.250 (0.854–1831)	0.252	1.325 (0.874–2.009)	0.185	1.030 (0.619–1.713)	0.909	1.005 (0.589–1.717)	0.984	1.377 (0.755–2.510)	0.297	1.767 (0.870–3.590)	0.115
Diabetes	1.823 (1.313–2.530)	< 0.001	1.709 (1.202–2.429)	0.003								
Current smoking	0.820 (0.578–1.165)	0.268	0.852 (0.586–1.238)	0.400	0.750 (0.445–1.266)	0.282	0.809 (0.470–1.394)	0.446	0.884 (0.547–1.429)	0.616	0.906 (0.537–1.530)	0.712
Hypercholesterolemia	1.838 (1.227–2.754)	0.003	1.847 (1.196–2.852)	0.006	1.973 (1.076–3.619)	0.028	1.830 (0.951–3.522)	0.071	1.515 (0.877–2.615)	0.136	1.642 (0.913–2.955)	0.098
Body mass index (kg/m2)	1.032 (0.980–1.085)	0.232	1.035 (0.980–1.094)	0.212	0.985 (0.914–1.062)	0.701	1.010 (0.933–1.093)	0.807	1.071 (1.000–1.147)	0.050	1.057 (0.980–1.140)	0.150
Glomerular filtration rate (ml/min)	0.994 (0.986–1.003)	0.186	0.999 (0.990–1.007)	0.790	0.999 (0.986–1.012)	0.902	1.002 (0.988–1.015)	0.817	0.990 (0.979–1.001)	0.084	0.996 (0.985–1.007)	0.480
LVEF (%),at baseline	0.975 (0.954–0.997)	0.026	0.972 (0.949–0.995)	0.017	0.991 (0.959–1.025)	0.605	0.986 (0.952–1.021)	0.419	0.971 (0.941–1.001)	0.055	0.967 (0.936–1.000)	0.048
Severity of coronary artery disease	1.277 (1.017–1.604)	0.035	1.272 (0.996–1.624)	0.054	1.414 (1.019–1.961)	0.038	1.363 (0.967–1.920)	0.077	0.981 (0.708–1.358)	0.907	1.010 (0.706–1.445)	0.956
Complete revascularization (%)	1.840 (1.275–2.655)	0.001	1.788 (1.203–2.657)	0.004	2.604 (1.595–4.252)	< 0.001	2.540 (1.511–4.269)	< 0.001	1.310 (0.730–2.352)	0.365	1.216 (0.631–2.341)	0.559
Collateral Rentrop class	1.448 (1.040–2.016)	0.028	1.459 (1.022–2.082)	0.037	2.013 (1.226–3.304)	0.006	2.156 (1.279–3.634)	0.004	0.863 (0.541–1.375)	0.534	0.827 (0.496–1.380)	0.468

MACCE: major adverse cardio-cerebral events; LVEF: left ventricular ejection fraction

Percent event-free from MACCE and repeat revascularization in non-diabetic and type 2 diabetic patients (A and B) and in those with good and poor coronary collateral circulation (C and D). MACCE: major adverse cardio-cerebral events

## Discussion

The results of this prospective, observational study show that: (1) T2DM was associated with reduced coronary collateralization; (2) successful revascularization of CTO lesions slightly improved left ventricular function; (3) there was no distinction between T2DM and non-diabetic effects of collaterals on MACCE and repeat revascularization after successful recanalization of CTO.

Notably, all patients in this study were specially selected as they received successful CTO-PCI and were encouraged to take guideline-recommended medications after the procedure. Our findings support the notion that T2DM represents an increased risk for CTO [2, 12, 13] and is a powerful independent factor for increased cardiovascular mortality and repeat revascularization after CTO-PCI [10, 11]. Abundant evidence has demonstrated that T2DM exerts a detrimental effect on glucose and lipid metabolism and vascular endothelial function, leading to development and progression of coronary atherosclerosis and unfavorable clinical outcomes [34–37]. In this study, T2DM was the only independent risk factor for poor coronary collateralization, suggesting that presence of T2DM is correlated negatively with the development of functional collateral arteries [24, 25], and may contribute partially to adverse prognosis of CTO patients [38]. Although presence of a chronic totally occluded lesion has been considered as a prerequisite for spontaneous collateral recruitment, the mechanism of collateral vessel growth is complex in situations where atherosclerosis affects large conductance arteries [39], and even becomes more complicated by the presence of T2DM in which multiple biochemical and cellular components are involved [25, 40, 41].

Another finding of this study is that around one-year post successful CTO-PCI, although patients with good coronary collateralization experienced a significantly lower rate of composite MACCE and repeat revascularization compared to those with poor collateralization, and Kaplan–Meier curves revealed the cumulative survival free from repeat revascularization and MACCE was significantly different according to the Rentrop class of coronary collateralization, multivariable Cox proportional hazards regression models revealed that the status of coronary collateralization was independently associated with long-term clinical outcomes only in non-diabetic patients but not in those with T2DM. The explanation for these observations is likely to be multi-factorial. Although the rate of MACCE and repeat revascularization was higher in T2DM group, there were more women, hypertension and multivessel vessel disease and lower baseline LVEF as well as poorer collaterals in type 2 diabetic patients. Numerous studies have shown that when the proximal part of a coronary artery is occluded, collateral circulation could, at least partially, supplies the downstream perfusion area via the arteriolar connection, thereby preventing or alleviating ischemia, and its extent is a primary determinant of the severity of myocardial damage (infarct size and/or left ventricular function) and mortality after transient or permanent coronary obstruction [14, 15**]**. However, blood supply of the well-developed collaterals may not fully substitute normal coronary flow [1, 42], and thus good collateral circulation for myocardial protection is not sufficient. Importantly, good collaterals in majority of patients will be rapidly subside after successful recanalization of a CTO, as antegrade blood flow is re-established and resistance is increased in the collateral vessels [38, 42]. Kim et al. found that good collateral circulation for myocardial protection only exists before recanalization forms an effective perfusion [43]. In addition, previous studies suggest that the occurrence of in-stent restenosis after successful CTO-PCI is not influenced by coronary collateralization [32] or even elevated in patients with good collaterals [44]. These could lead to a similar or increased rate of repeat revascularization and overall MACCE in patients with good collaterals after successful CTO-PCI.

In this study, mean LVEF was slightly increased by 2.50% during follow-up which is lower than that previously reported (4.44%) [45]. Although patients with T2DM did have lower LVEF at baseline, the response to CTO-PCI was similar to non-diabetics and similarly there was a trend to better response to LVEF in those with good coronary collaterals. Nevertheless, non-diabetic patients with good collateralization had a significantly greater improvement in left ventricular function compared to those with poor collaterals. Collaterals are associated with improved LVEF (maintained viability to the CTO) and appear to be prognostically beneficial, although there were some trials that have demonstrated otherwise. The present study is confirmatory. It is also known that coronary collateralization is reduced in patients with T2DM [24, 25]. This may be due to more multivessel disease affecting donor artery perfusion pressure and also differences in biomarkers that stimulate collateral development in diabetics [39]. The reduction in target vessel revascularization may be related to improved viability in those with collaterals maintaining brisk flow as supply matches demand. Ripley et al. found that viable myocardium was present in 83% of patients with good collaterals versus 38% of those with poor collaterals [46]. Likewise, Choi et al. observed that increased angiographic collateral flow was associated with lower degree of late gadolinium enhancement transmurality after CTO recanalization, in contrast, poorly collateralized myocardial segments would be less likely to recover function in comparison with well collateralized segments [47]. Compared with non-diabetics, type 2 diabetic patients had lower LVEF at baseline, and the change in LVEF was smaller and did not differ after successful CTO-PCI irrespective of collateral status. These findings are consistent with previous reports that myocardial and collateral function is more severely depressed in the diabetic setting [10, 11, 24, 25].

### Study limitations

We recognize that there are several limitations in our study. First, a potential weakness of our study is the small sample size (especially the number of patients with T2DM was low). Both T2DM and non-diabetic groups seem to behave similarly and the magnitude of the effects observed in diabetics and non-diabetics was also similar in size. Therefore, no significant difference between patients with good collaterals and those with poor collaterals for T2DM group may be simply caused by under-powering. Second, the patient distribution is heterogeneous as about 80% of the patients were male. Third, the study is cross-sectional for the point of coronary collateral and MACCE or improvement of LVEF investigation, thereby allowing us to detect association, not to formulate causal link. Fourth, the presence and degree of coronary collaterals were evaluated according to the Rentrop scoring system. Although this angiographic assessment of coronary collaterals is easily to be incorporated into the routine clinical practice, coronary collaterals may be more accurately assessed by collateral flow index with simultaneous measurement of aortic pressure and the distal pressure within the occluded segment of the culprit coronary artery [42]. In addition to the morphological evaluation, in diabetic patients with a well-known microvascular damage it would be essential to have a functional evaluation of the coronary ischemia. Finally, clinical follow-up was relatively short in duration, which may result in limited number of MACCE particularly for hard endpoints (e.g., death), and amount of viable myocardium or ischemia was not evaluated using functional tests, thus large-scale prospective randomized studies are required to further determine the impact of coronary collateral circulation on prognosis after successful CTO-PCI.

## Conclusions

The present study suggests that presence of T2DM is associated with reduced coronary collateralization. The effects of the status of coronary collateralization on long-term clinical outcomes and left ventricular function appear to be similar in size in type 2 diabetic and non-diabetic patients after successful recanalization of CTO. These findings may provide clinical insight into the management of patients with stable coronary artery disease.

## Supplementary information

**Additional file 1: Table S1.** Predictors of MACCE and repeat revascularization in all patients on univariable and multivariable analysis.

**Additional file 2: Table S2.** Predictors of MACCE and repeat revascularization in patients without T2DM on univariable and multivariable analysis.

**Additional file 3: Table S3.** Predictors of MACCE and repeat revascularization in patients with T2DM on univariable and multivariable analysis.

## Data Availability

Data generated or analyzed during this study are included in this published article.

## Abbreviations

CTO: Chronic total occlusion
HR: Hazard ratio
LVEF: Left ventricular ejection fraction
MACCE: Major adverse cardio-cerebral events
NYHA: New York Heart Association
PCI: Percutaneous coronary intervention
T2DM: Type 2 diabetes mellitus
SD: Standard deviation
